# Campus source to sink wastewater surveillance of severe acute respiratory syndrome coronavirus-2 (SARS-CoV-2)

**DOI:** 10.1016/j.crmicr.2024.100240

**Published:** 2024-05-06

**Authors:** M. Folkes, V.M. Castro-Gutierrez, L. Lundy, Y. Bajón-Fernández, A. Soares, P. Jeffrey, F. Hassard

**Affiliations:** a Cranfield University, College Road, Cranfield, Bedfordshire, MK43 0AL, UK; b Center for Research on Environmental Pollution (CICA), Universidad de Costa Rica, Montes de Oca, 11501, Costa Rica; c Department of Natural Sciences, Middlesex University, NW4 4BT, UK; d Institute for Nanotechnology and Water Sustainability, College of Science, Engineering and Technology, University of South Africa, Johannesburg, South Africa

**Keywords:** Wastewater surveillance, Wastewater-based surveillance (WBS), Wastewater-based epidemiology (WBE), Surveillance COVID-19, SARS-CoV-2, Coronavirus, Pepper mild mottle virus (PMMoV)

## Abstract

Wastewater-based surveillance (WBS) offers an aggregate, and cost-effective approach for tracking infectious disease outbreak prevalence within communities, that provides data on community health complementary to individual clinical testing. This study reports on a 16-month WBS initiative on a university campus in England, UK, assessing the presence of SARS-CoV-2 in sewers from large buildings, downstream sewer locations, raw wastewater, partially treated and treated effluents. Key findings include the detection of the Alpha (B.1.1.7) variant in wastewater, with 70 % of confirmed campus cases correlating with positive wastewater samples. Notably, ammonium nitrogen (NH_4_-N) levels showed a positive correlation (ρ = 0.543, *p* < 0.01) with virus levels at the large building scale, a relationship not observed at the sewer or wastewater treatment works (WWTW) levels due to dilution. The WWTW was compliant to wastewater standards, but the secondary treatment processes were not efficient for virus removal as SARS-CoV-2 was consistently detected in treated discharges. Tools developed through WBS can also be used to enhance traditional environmental monitoring of aquatic systems. This study provides a detailed source-to-sink evaluation, emphasizing the critical need for the widespread application and improvement of WBS. It showcases WBS utility and reinforces the ongoing challenges posed by viruses to receiving water quality.

## Highlights

• Wastewater based surveillance (WBS) offers cost-effective infectious outbreak tracking at campus community level.
• Alpha variant detected in wastewater, but not aligning with campus cases.
• Impacts of public health restrictions evident from viral detection in wastewater.
• SARS-CoV-2 gene fragments not removed efficiently within wastewater treatment works (WWTW).
• Ammonium levels correlate with SARS-CoV-2 in buildings, less significant in sewer or WWTW.

## Graphical abstract

**Figure:**
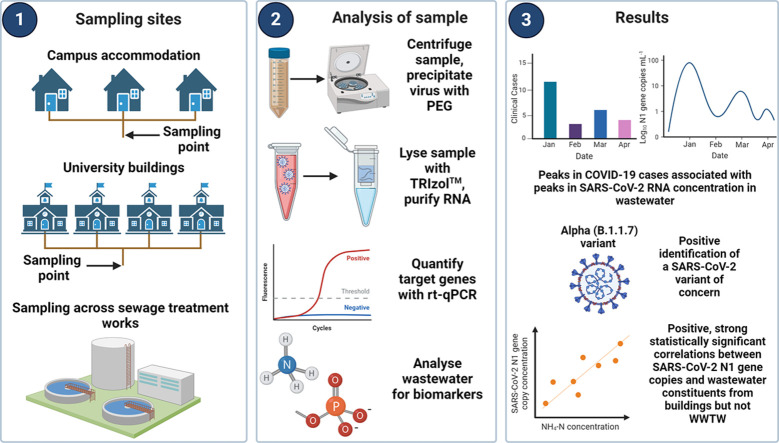

## 1 Introduction

Operating universities safely during the COVID-19 pandemic posed significant logistical, political, and public health challenges. Within university settings, the close proximity of students, mixed vaccination rates, and frequent gatherings in confined, poorly ventilated spaces exacerbated these challenges. In the UK, the impact was pronounced during the second and third COVID-19 waves, dominated by the Alpha and Delta variants. In the UK, during the academic years 2019–2020 and 2020–2021, the majority of students were under 30 years of age (Higher Education Statistics Agency, 2023). Due to the initially limited supplies of vaccines, the majority of people aged 30 and under with no known existing health conditions were ineligible for vaccination until June 2021 (Rough, 2023). Consequently, only 20 % of people aged under 40 had received at least one dose of a COVID-19 vaccine by the end of June 2021 (Public Health England, 2021d). Thus, the majority of students were without vaccine protection for much of the pandemic. Identifying and mitigating transmission hotspots on campus emerged as a critical concern for university administrators and safety personnel. Strategies employed included online lectures, hybrid learning models, and mandatory testing and isolation protocols as logistical and public health measures to mitigate risks (Betancourt et al., 2021).

Nevertheless, in-person instruction remained crucial for the financial viability of universities and is vital for the social, emotional, and educational well-being of students (Bork-Hüffer et al., 2021). The shift to online learning prompted by university closures has resulted in increased student dissatisfaction, and significant financial repercussions for both institutions and students and lost learning opportunities (Bolton and Hubble, 2021). Moreover, students from lower-income backgrounds faced heightened risks of financial and emotional distress due to COVID-19 restrictions, exacerbating socioeconomic disparities and widening the educational achievement gap associated with family income (Bligh et al., 2021). Non-pharmaceutical public health measures, including symptom screening, clinical testing, double masking, reduced class sizes and enhanced ventilation (Castro-Gutierrez et al., 2022) were implemented on university campuses to curb viral spread (Brooks-Pollock et al., 2021). Such interventions were crucial for maintaining access to higher education, advancing health equity, minimizing morbidity and mortality, and preventing the rise of new variants of concern (VoCs). Timely detection and interruption of transmission pathways are particularly vital in scenarios of low infection rates and in the face of more transmissible variants (Harvey et al., 2021). Likewise, expansive communal environments like prisons, dormitories and cruise ships can amplify infectious diseases like COVID-19, where crowding and poor ventilation can worsen transmission risks (Nowotny et al., 2021).

Wastewater based surveillance (WBS) has been used as a tool for passive surveillance of community, building, and individual level SARS-CoV-2 infections in municipalities (Crits-Christoph et al., 2021), schools (Hassard et al., 2021), hospitals and care homes (Gallardo-Escárate et al., 2021), ports (Ahmed et al., 2020a), prisons (Hassard et al., 2023) and universities (Karthikeyan et al., 2021a). WBS is defined as the retrieval of human health information from wastewater through the analysis of specific chemicals or human metabolites, excreta or disease linked products (Castiglioni et al., 2014). WBS provides aggregate and anonymous samples of populations contributing to a sewershed, therefore informed consent (of individuals) is usually not required. However, oversight from ethical boards is recommended. WBS has been used for monitoring population-level infection trends and the spread of variants at a sewershed level, but WBS is unlikely to completely replace clinical testing for infectious diseases (Tlhagale et al., 2022), as the two approaches generate distinct data - with WBS providing prevalence and clinical testing primarily incidence. Both datasets can be used for longitudinal studies on disease outbreaks and dynamics of transmission, however WBS does this for a fraction of the cost of clinical tests. One study from a University in the USA indicated that the costs of clinical sampling were $17.5 per person compared to an equivalent WBS of $0.30 per person tested (Wright et al., 2022). This implies significant cost reductions, particularly valuable in resource-constrained settings or when surveillance expands to monitor multiple pathogens concurrently. It is also crucial for comprehensive analyses like antimicrobial resistance, where broad-spectrum or diverse target monitoring is necessary (Choi et al., 2018).

WBS has proven effective for disease monitoring (e.g., for COVID-19 infections), as viral RNA from SARS-CoV-2 is present in the faeces of infected individuals allows for the surveillance of COVID-19 through the quantification of the number of copies of specific genes in the sewage (Medema et al., 2020). Despite concerns over potential SARS-CoV-2 infections from faecal aerosols, a systematic review found no strong evidence of human SARS-CoV-2 faecal-oral transmission (Termasen and Frische, 2023). However, several uncertainties persist. These include, but are not limited to, issues related to: sample storage (Ahmed et al., 2020b), contamination with RT-qPCR inhibitors in wastewater (Ahmed et al., 2022), dilution effects in combined sewer systems (Sims and Kasprzyk-Hordern, 2020), and potential underrepresentation of disease prevalence due to paucity of clinical data. Furthermore, the optimal scale for sampling to maximize effectiveness remains undetermined, as does the need to account for variables such as defecation frequency and movement patterns of persons within and between sewersheds. Despite these challenges, evidence strongly supports WBS traditional public health surveillance for prevalence determination of population level infections (Hart and Halden, 2020). The application of WBS on university campuses for monitoring of COVID-19 infections is well-documented. Near-source sampling enables the detection of infected individuals within specific buildings or groups of buildings. This facilitates targeted clinical testing and case isolation (Corchis-Scott et al., 2021), and informs health and safety policies (Karthikeyan et al., 2021b). WBS studies have demonstrated statistically significant correlations between SARS-CoV-2 gene copy numbers in wastewater and clinically confirmed cases on campuses (Wright et al., 2022). Also, the ability to detect SARS-CoV-2 variants in wastewater (Vo et al., 2022) offers pre-emptive measures against potential outbreaks and aiding in the management of public health resources efficiently, for example medicine prioritisation (Boehm et al., 2022), although high-fidelity sampling is critical (Karthikeyan et al., 2021b). WBS has also shown promise for monitoring emerging viral threats (Tisza et al., 2023).

However, there is a notable gap in tracing SARS-CoV-2 from its source through the conveyance system to treatment and eventual environmental discharge especially in contiguous wastewater conveyance systems. Comprehensive data establishing the variability in virus removal across different scenarios and treatment processes are needed to enhance understanding of how WBS's can improve equitable access to sufficient public health monitoring for informed decision-making.

Accordingly, the purpose of this study was to (i) monitor wastewater effluent streams at the Near-Source, In-Sewer and WWTW level in a geographically isolated, single-source sewershed across a 16-month period for the presence and abundance of SARS-CoV-2 genetic material; (ii) investigate if SARS-CoV-2 gene copy data can be linked to the number of clinically confirmed cases on the campus/in the local community and (iii) to assess the efficacy of the WWTW at removing SARS-CoV-2 gene copies from the wastewater prior to environmental discharge.

## 2 Materials and methods

### 2.1 Study site

**Fig. 1:**
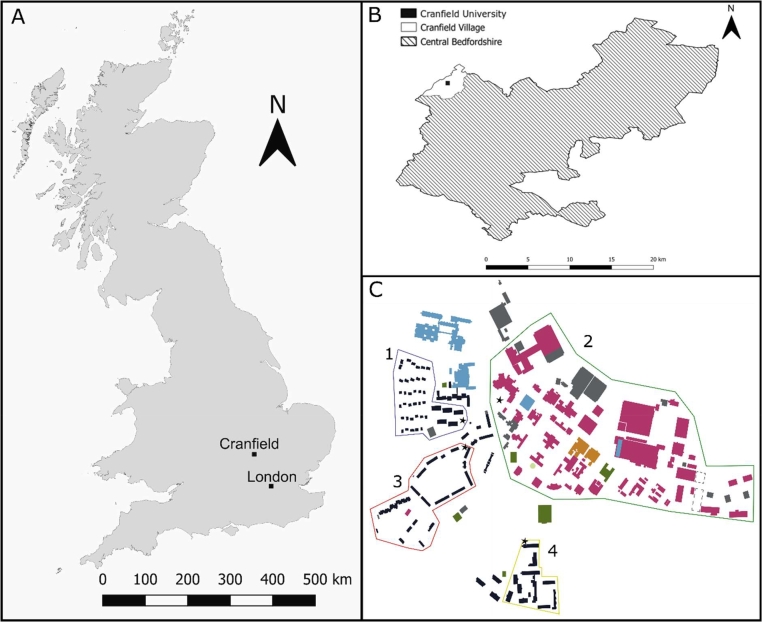
A – Cranfield in relation to London; B – Cranfield University in Cranfield Village within Central Bedfordshire Lower Tier Local Authority; C – (1) Halls of Residence A, (2) Technical In-Sewer, (3) Residential In-Sewer, (4) Halls of Residence B.

Cranfield University, a postgraduate institution situated in Bedfordshire, England (Fig. 1A-C), spans circa 59 significant buildings. It hosts a median residential population of 1200, augmented by a transient weekday population of 3800 staff and students, based on 2018–2019 figures. Weekend populations decrease to approximately 1300. The COVID-19 pandemic prompted the development of various public health measures nationally across England, which were fully implemented within Cranfield University's campus, significantly influencing the dynamics of the campus population. Cranfield University has an independently operated and self-contained sewer system which receives primarily wastewater from the university technical buildings, halls of residence and residential properties. This sewershed conveys wastewater from large buildings including residences and campus businesses, and university estate. This sewershed does not contain industrial inputs and so represents a quite novel and unbiased case study in terms of the absence of significant wastewater signal dilution effects, something rarely reported in WBS studies. A proportion of Cranfield University's wastewater is combined with rainfall and surface water drainage, similar to most wastewater conveyance schemes in high-income countries. In these systems, the proportion of the sewer network which is municipal only versus combined remains difficult to quantify without accurate sewer maps, models or flow monitoring. Here, campus wastewater was sampled in two types of location, from sewers collecting from individual buildings or at external sewer access points collecting water from a sewer fed by numerous buildings. At external sewer access points, there was potential dilution from other water sources, such as industrial scale laundries, not likely to contain the faecal viral signals especially in grey water from laundry rooms, showers, and other facilities.

The wastewater system employs gravity to convey effluent to a central pumping station, subsequently directing it to the WWTW. This facility, designed to handle a population equivalent (P.E) of 3278 ± 914 in 2018, processes the entirety of the campus's daily wastewater. The average daily flow rates were recorded as 465.50 ± 129.82 m³ *d*^−1^ in 2018, slightly decreasing to 459.35 ± 137.88 m³ *d*^−1^ in 2019, and further to 449.04 ± 163.74 m³ *d*^−1^ in 2020. This trend in wastewater flow aligns closely with the fluctuations in campus population, as evidenced by a significant positive correlation (Spearman's Rank; ρ(186) = 0.673, *p* < 0.01). Sampling was conducted at three spatial scales “Near-Source,” “In-Sewer,” and at the WWTW - to detect and monitor potential COVID-19 infections (Fig. 1C). The hypothesis guiding this study was that it would be possible to distinguish and attribute infections both between and within these distinct sewer catchments using WBS. Sampling frequency was adapted in response to local and national lockdowns, which accordingly restricted access to sites and also prevented significant clinical testing of students onsite. Thus, WBS provided an opportunity to learn more about infection dynamics during data dearth from other public health sources such as individual clinical testing.

### 2.2 Sample locations

Herein, we targeted sewer access points that enabled sample collection across diverse on-campus population groups. However, higher frequency WBS sampling and clinical confirmation remains the ‘gold standard’ for environmental monitoring efforts (Fielding-Miller et al., 2023). During the study, an anomaly observed was students spending around 65 % of their time in isolation, engaging in remote learning. This deviates from typical campus activity, characterized by frequent movement and interaction across different areas and sewersheds.

The sewersheds sampled for this investigation included "Hall of Residence A" and "Hall of Residence B" (Fig. 1C), designated as Near-Source sampling points. These points are located in sewer access areas servicing two student halls, collectively housing 57 % of the student residence population. A third sewershed, “Residential Houses In-Sewer”, was identified as a Sewer-Scale sampling point, positioned downstream of multiple sewer pipes serving a mix of shared student accommodations and individual family homes. Another sampling point, "University Technical Buildings In-Sewer," represented a Sewer-Scale sampling location situated downstream of several sewer pipes that cater to the university's technical facilities. This comprehensive selection of sampling points was chosen to provide a representative analysis of the wastewater generated by distinct segments populations of the university community (Fig. 1C).

The Cranfield Campus WWTW was the focus of an intensive sampling campaign aimed at evaluating its effectiveness in removing SARS-CoV-2 RNA fragments across the WWTW and evaluating the removal efficacy of each treatment stage. Sewage treatment at the Cranfield Campus WWTW is a multistage process, consisting of: a screen to remove coarse material from the waterwater, a flow equalization tank (the “balance tank”) that also acts as a settlement tank, a set of primary lamella clarifiers (the “primary lamella”), a trickling filter (the “roughing filter”), a second set of lamella, a set of two trickling filters (the “secondary trickling filter”), and the final effluent where the treated sewage is discharged into a small river called Chicheley Brook. The flow equalization tank settles solids by slowing water flow, allowing gravity to facilitate sedimentation. Water from the base of lamella clarifiers is pumped at an upflow rate lower than the particles' settling velocity, enhancing sedimentation. The primary clarifier removes sufficient particles to prevent overloading the roughing trickling filter, maintaining its BOD reduction efficiency. The secondary clarifier settles sloughed biosolids. Trickling filters, containing plastic matrix media encouraging a biofilm to develop for BOD removal or NH_3_ transformation. The Cranfield Campus WWTW does not have an activated sludge tank, and the effluent does not require disinfection prior to its release to the environment. The points sampled were the WWTW influent after settling in the flow-equalisation tank, the post-primary lamellas (after the primary lamellas), post-roughing trickling filter (after the first tricking filter), post-secondary trickling filter outlets and the final effluent. Regular samples were routinely obtained from the 19/03/2020 to the 27/07/2021 from the influent to the WWTW after it had settled in the flow equalisation tank. Sampling from the other four sampling points began on the 22/01/2021 and ended on the 27/07/2021.

The flow equalisation tank captures a representative sample of wastewater from the entire campus population. The median residence time in the flow equalisation tank was recorded at 15.34 h (with a standard deviation of ± 5.46) throughout the study, offering a representation of the campus's wastewater profile. The flow equalisation tank underwent cleaning and desludging processes on a weekly basis to maintain optimal functionality.

### 2.3 Sample collection

In this study, a total of 488 wastewater samples were analysed for the presence of SARS-CoV-2, of which 284 were processed in duplicate and 204 as single samples. Additionally, 377 of these samples underwent further examination to assess various wastewater characteristics. Among these, 112 samples, representing 39.7 % of the total, tested positive for the N1 gene indicative of SARS-CoV-2 presence. Concurrently, the study quantified the abundance of Pepper Mild Mottle Virus (PMMoV) in 108 samples that demonstrated a positive detection for SARS-CoV-2. PMMoV, a plant pathogen prevalent in human faeces, serves two roles in wastewater virology studies: firstly, as a population equivalent normalising agent SARS-CoV-2 data in municipal wastewater due to its widespread presence (D'Aoust et al., 2021), and secondly, as an indicator of the adequacy of the viral extraction process by verifying if the tracer levels fall within expected ranges for municipal wastewater (Symonds et al., 2018; Ahmed et al., 2022).

In the initial phase of the study, from the 19/03/2020 to the 15/01/2021, weekly 500 ml grab samples were collected from the WWTW inlet and subsequently stored at -80 °C within two hours to preserve the samples for future analysis. From the 22/01/2021, weekly 1 L grab samples were collected from the aforementioned stages of the WWTW. Sampling from the campus sewersheds began on the 22/01.2021. Aquacell P2-COMPACT autosamplers (Aquamatic, UK) were used to collect samples from the sewer access points across the campus. Samples were taken from 7:00am on one day to 7:00am the following day. These devices were programmed to collect a fraction of the wastewater stream, precisely 16.6 ml every five minutes, culminating in approximately 200 ml per hour, and to automatically store the collected sample in an integrated 5 L high-density polyethylene (HDPE) collection vessel which was refrigerated. This yielded a 4.8 L composite sample over the 24 h sampling period. Samples were collected from the reservoir between 7:00am and 8:00am. Post-collection, the samples were thoroughly mixed, and a 1 L aliquot was reserved for further analysis in a 1 L polypropylene bottle.

From 22nd of January 2021 onwards, additional 1 L grab samples were systematically collected from various stages of the WWTW process, including the inlet flow, post-primary lamella, post-roughing trickling filter, post-secondary trickling filter outlets, and the final effluent on a weekly basis. All samples were transported back to the laboratory on melting ice and were immediately refrigerated at a temperature ranging from 2.5 °C to 4 °C. Three 200 ml aliquots of each sample were taken: the first for RNA extraction, the second for cryogenic preservation at -80 °C, and the third for analysing wastewater constituents. The RNA extraction and cryogenic storage processes were conducted on the day of collection, while the constituent analysis was completed within 48 h.

### 2.4 RNA extraction

To optimize the quality and concentration of RNA and enhance the recovery of SARS-CoV-2, methodological refinement was conducted. The first extraction method, referred to as Protocol 1, was derived with modifications from the procedure outlined by Farkas et al. (2021). In brief, the method involved aliquoting a 200 mL sample into four 50 mL falcon tubes for initial centrifugation. The supernatant was transferred to new containers, and concentrated using polyethylene glycol (PEG) precipitation with a murine norovirus spike as an extraction control. The samples were centrifuged to form a pellet, excess PEG and supernatant were discarded, and the resultant pellet was resuspended in phosphate buffer saline (PBS) for RNA extraction using the NUCLEISENS® kit on a MINIMAG® system.

Subsequently, Protocol 2 was developed, incorporating adjustments from the technique outlined in Amirouche et al. (2021). This second protocol employed Trizol-chloroform to separate RNA from retained wastewater constituents (e.g., cellular proteins) and DNA prior to the RNA purification step, significantly improving resultant RNA yield and quality. Protocol 2 involved a two-step process for RNA extraction and purification from wastewater samples. Samples were first centrifuged to remove solids from the wastewater, followed by PEG precipitation spiked with a Φ6 virus as an extraction control. After shaking and refrigerating, the samples were centrifuged to concentrate the viral particles, which were then lysed using TRIzol™ and chloroform for RNA isolation. The RNA was then purified using a Macherey Nagel Nucleospin RNA kit.

The initial set of 99 samples, collected between the 22/01/2021, and the 03/03/2021, were processed using Protocol 1. Following this, Protocol 2 was applied to samples numbered 100 to 389, beginning on the 04/03/2021, to the 21/072021. Detailed descriptions of the SARS-CoV-2 RNA extraction and purification methods for each protocol are available in Supplementary Material 1.

### 2.5 Quantification with RT-qPCR

Post-extraction, RNA concentrations in samples were quantified using the Qubit™ system (Fisher Scientific, UK). Samples with RNA concentrations exceeding 100 ng/mL were diluted to a minimum of 1:10 with RNase-free sterile water to meet the analysis criterion of less than 100 ng/mL, ensuring optimal process efficiency. SARS-CoV-2 RNA was detected through RT-qPCR employing the UltraSense™ One-Step Quantitative RT-PCR System (Thermo Fisher, UK), targeting both the nucleoprotein (N1) and envelope (E) protein gene fragments. This selection was based on their specificity and stability, minimizing the likelihood of SARS-CoV-2 misidentification or false negatives. RNA samples were analysed in duplicate, with nuclease-free water serving as negative controls, following procedures outlined by Castro Gutierrez et al. (2022). Quantification involved comparing cycle threshold (CT) values against an external standard curve derived from synthesized plasmids containing target sequences. Positive SARS-CoV-2 identification was based on N1 gene copy numbers surpassing the defined limit of detection (LOD), with LOD values for Protocols 1 and 2 established as per Castro Gutierrez et al. (2022). Specifically, LODs for N1 and E gene fragments were 1268 GC/L and 2968 GC/L for Protocol 1, and 956 GC/L and 2401 GC/L for Protocol 2, respectively. A sample was considered positive for SARS-CoV-2 if the N1 gene concentration exceeded these LOD values. Negative samples were recorded with an N1 gene concentration at half the LOD value to ensure a conservative approach to detection limits.

A total of 108 previously extracted samples were evaluated for the presence of PMMoV. The quantification of PMMoV was carried out using a modified method from Jafferali et al. (2021), tailored to align with the capabilities of the available instrumentation. The assay employed forward and reverse primer sequences as suggested by Jafferali et al. (2021), along with custom TaqMan probes (ThermoFisher, UK) for the specific detection of PMMoV gene targets. 5 µL of each sample was analysed under the following thermal cycling conditions on a QuantStudio™ 7 Pro-Real-Time PCR System (ThermoFisher, UK): an initial hold at 55 °C for 60 min, denaturation at 95 °C for 5 min, followed by 45 cycles of denaturation at 95 °C for 15 s, annealing/extension at 60 °C for 1 min, and final extension at 65 °C for 1 min. To ensure assay integrity, negative controls were included in every batch. Quantification was based on the comparison of Ct values against an external standard curve derived from commercially synthesized plasmids that contain the target PMMoV sequence. A sample was determined to be positive for PMMoV when its Ct value was detected within 45 cycles.

### 2.6 Wastewater constituents

Wastewater samples were analyzed for total suspended solids (TSS, Method 2540 D), ammonium (NH_4__—_N, Method 4500-NH3 F), orthophosphate (PO_4_-P, Method 4500-P E), total chemical oxygen demand (tCOD, Method 5220 D), pH (Method 4500-*H* + *B*), conductivity (Method 2510 B), and oxidation–reduction potential (ORP, Method 2580 B) following the 'Standard Methods for the Examination of Water and Wastewater (APHA, 2017)’ guidelines**.**

### 2.7 Normalization

Normalizing SARS-CoV-2 concentrations in wastewater to chemical or biological markers is a strategy employed to estimate the size of the contributing population. Among the suggested biological markers for this purpose, Pepper Mild Mottle Virus (PMMoV) has been highlighted for its utility (D'Aoust et al., 2021). Beyond population estimation, PMMoV serves as a benchmark for assessing the efficiency of RNA and DNA extraction processes (Ahmed et al., 2022). In this study, the gene copy data were normalized to a marker using an equation modified from Sweetapple et al. (2022), facilitating a more accurate interpretation of the viral load in relation to the population size. Where the concentration of SARS-CoV-2 gene targets (*GC*) and the concentration a specific biomarker are both known (*X*), the concentration of gene copies can be normalised to a specific biomarker (*GC_n_*) using Eq. (1).

(1) $$GC_{n}\;=\;\frac{GC}{X}$$

### 2.8 SARS-CoV-2 VoC identification

SARS-CoV-2 VoCs was conducted using the Artic multiplex PCR technique for sequencing the SARS-CoV-2 genome (Tyson et al., 2020) executed by Eurofins (Germany). This method identifies mutations indicative of specific viral lineages, impacting functional traits such as virus transmissibility and infectivity. From the positive SARS-CoV-2 wastewater samples, ∼4 % were selected for sequencing. Of these, only two samples reached the necessary genome coverage for in-depth analysis, while thirteen samples did not achieve sufficient coverage across vital genes for further examination. Raw sequence reads were processed through the *ncov2019-artic-nf v3* pipeline (https://github.com/connor-lab/ncov2019-artic-nf). Subsequently, VarScan was utilized to detect Single Nucleotide Polymorphisms (SNPs) and insertions/deletions (Indels), which were then screened against the signature mutations of currently circulating VoCs using a custom script. The establishment of SARS-CoV-2 VoC profiles was guided by designations from Public Health England (https://github.com/phe-genomics/variant_definitions), allowing for the categorization of wastewater samples based on the presence of specific SNPs as follows:

1. B.1.1.7 (VOC-20DEC-01) Alpha Variant**:** Confirmation of this variant required the detection of ≥10 out of 13 signature SNPs. It was considered possible with ≥5 detected SNPs, and not detected if ≤4 SNPs were found.
2. B.1.617.2 (VUI-21APR-02) Delta Variant: This variant was confirmed if ≥9 of 13 signature SNPs were found, deemed possible with ≥5, and not detected with ≤ 4.
3. Delta Substrains (AY.43, AY.46.5, and AY.2): A confirmed status was assigned with ≥70 % of the SNPs detected, possible if ≥50 % were detected, and not detected if ≤50 %.

Samples were classified as "Confirmed" when there was strong evidence of a variant's presence, and as "Possible" when there was some indication of a variant. To further explore the transmission dynamics of SARS-CoV-2 in the community, logistic regression was employed to examine the relationship between the detection levels of N1 and E gene copies per millilitre in wastewater (as predictor variables) and the likelihood of identifying a VoC (as the response variable). Additionally, multinomial logistic regression was used to assess the relationship between the concentrations of N1 and E gene copies in wastewater samples and the identification of a sample as either Alpha or Delta variant, or as not detected, according to the outlined criteria.

### 2.9 Statistics

The dataset underwent statistical analysis using SPSS (Version 28, IBM, 2021) for analytical procedures, while Microsoft Excel (Version 2108, Microsoft, 2021) served to organize and manage the comprehensive database of samples. This database facilitated the identification of correlations between the occurrences of SARS-CoV-2 positive detections in wastewater samples, both preceding and following confirmed cases. Additionally, bootstrapping techniques were applied to compute bias-corrected confidence intervals (CI), established at a 95 % confidence level.

### 2.10 Other data sources

Clinical case data for the campus was provided on an approximately daily basis by the Cranfield Site Access Group, a local decision administration body designed to safeguard public health on campus. This data was supplemented by extracting information on cases within the lower-tier local authority area of the Central Bedfordshire Council from the national database on a weekly basis. This additional data was accessed through the UK government's official COVID-19 data portal, available at https://coronavirus.data.gov.uk/details/download, ensuring an overview of the local epidemiological situation – based on available data.

## 3 Results and discussion

### 3.1 Wastewater SARS-CoV-2 data

**Fig. 2:**
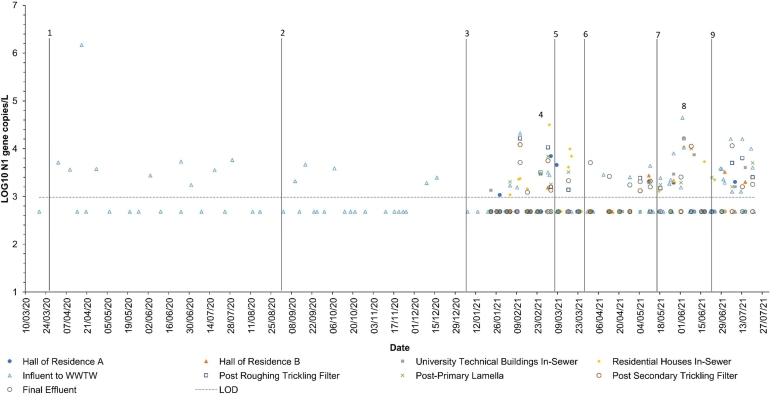
Wastewater N1 GC concentration at study sampling points. Numbers 1–3 represented a period of enhanced university and national restrictions. Numbers 4–9 represent a period of enhanced sampling due to change in university and national restrictions.

In the initial sampling phase (low frequency sampling, single sample point) from 19/03/2020 to 15/01/2021, SARS-CoV-2 levels in the influent of the WWTW ranged from 1.73 × 10^3^ to 1.48 × 10^6^ N1 GC/L. The peak wastewater abundance, observed on 20/04/2020 (Fig. 2, Table 1), was likely due to the dominance of the wild-type strain during local and national lockdowns, and widespread infections prior to national efforts to curb spread. The peak viral abundance detected via WBS on 20/04/2020, which subsequently decreased gradually provides insights into the dynamics of viral transmission and the immediate impact of national public health interventions (Table 1) and their probable effect on local infection dynamics. The viral fragments declined to between 10^3^ and 10^4^ GC/L from 27/04/2020 to 15/01/2021 (Fig. 2) suggesting a reduction in the virus spread on campus, potentially reflecting the effectiveness of identified control measures. This observed trend highlights the utility of WBS as a non-invasive tool for monitoring SARS-CoV-2 prevalence. In this instance, WBS was able to identify an increase in COVID-19 infections prior to their detection through clinical testing. Hence, WBS can be a useful tool to identify outbreaks of infections before they occur, assisting in the implementation and monitoring the efficacy of targeted containment strategies. WBS can be complimentary to traditional clinical tests, where testing capacity for clinical tests is constrained.

**Table 1:** England's national and university restrictions, VOC, campus population and LTLA vaccination rate. * (Public Health England, 2020)**, †** (Public Health England, 2021a)**, ‡** (Public Health England, 2021b)**, §** (Public Health England, 2021c)**.**

Number	Date	National restrictions	Campus restrictions	Dominant Variant of Concern (VOC)	Median people living on campus	LTLA Level of 1st vaccination (%)	LTLA Level of 2nd vaccination (%)
**1**	25/03/2020	Full national lockdown.	Access to campus restricted.	Undetermined*	977	0	0
**2**	01/09/2020	Limited restrictions; schools and universities open	Face-to-face teaching permitted.	Undetermined*	572	0	0
**3**	06/01/2021	National lockdown: essential services open.	Access to campus restricted.	Alpha-variant†	1175	1.73	0.36
**4**		No change.	No change.	Alpha-variant‡	1160	20.71	0.49
**5**	08/03/2021	No change.	Partial return to face-to-face teaching.	Alpha-variant§	1147	37.28	1.41
**6**	29/03/2021	Outdoor socialisation permitted in groups of 6.	No change.	Alpha-variant§	1147	56.21	3.85
**7**	17/05/2021	Most businesses re-open.	Full return to face-to-face teaching.	Delta-variant§	1081	64.75	32.92
**8**		No change.	No change.	Delta-variant§			
**9**	21/06/2021	Removal of all restrictions.	Campus access restrictions removed.	Delta-variant§	1013	82.61	64.15

During the second phase (low frequency sampling, encompassing campus and WWTW sample points) from 22/01/2021 to 27/05/2021, SARS-CoV-2 levels were found to be between 1.06 × 10^3^ and 2.60 × 10^5^ GC/L (Fig. 2). The highest concentration during this period, recorded on 02/03/2021 at the Residential In-Sewer point (Fig. 2, Table 1), coincided with the Alpha variant (B.1.1.7) becoming the dominant variant. This coincided with a national easing of lockdown restrictions. Concentrations steadily decreased from this peak, falling below the LOD by 14/04/2021. Median concentrations for campus and WWTW samples were 3.22 × 10^3^ GC/L and 2.93 × 10^3^ GC/L, respectively. This trend was identified retrospectively, as is the case in many WBS studies. This does, however, highlight the efficacy of the public health measures implemented at the time. Thus, WBS can be used to monitor the prevalence of COVID-19 infections as containment strategies are modified and for the identification of new and emerging variants coupled to the impact of public health restrictions.

The third phase (high-resolution WWTW influent analysis) from 04/05/2021 to 21/07/2021 showed SARS-CoV-2 levels ranging from 9.86 × 10^2^ to 4.42 × 10^4^ GC/L. The peak on 02/06/2021 at the WWTW influent point (Fig. 2) likely indicated the Delta variant (B.1.617.2) becoming the dominant strain on the campus during further relaxations of national and local lockdown measures (e.g., a full return to face-to-face teaching). However, this was not confirmed as few samples were able to be sequenced to identify the variant present in the samples. Following this peak, the SARS-CoV-2 viral fragments concentration decreased to 3.25 × 10^3^ GC/L by 01/07/2021, increased again to 1.61 × 10^4^ GC/L by 13/07/2021, and finally dropped below the LOD by 21/07/2021. Median values for SARS-CoV-2 gene copy concentration were consistent between the campus and WWTW sample points throughout this phase. In the third phase, focused on high-resolution analysis at wastewater treatment sites, SARS-CoV-2 levels demonstrated variability and a significant peak indicating the Delta variant's dominance during further easing of public health restrictions. Subsequent fluctuations in viral concentrations, ultimately decreasing below detection, mirrored public health interventions' impact nationally (Table 1) reflecting localised infection dynamics of immobile population within this single sewershed. Consistent median values across sampling points underscore the comprehensive monitoring's role in reflecting the virus's presence and guiding health measures amidst evolving pandemic conditions.

Fig. 2, alongside the metadata outlined in Table 1, illustrates the impact of adjustments in England's national and campus-specific restrictions on the concentration of SARS-CoV-2 N1 gene copies in wastewater samples. Markers 1–3, 5–7, and 9 indicate modifications to national and/or local restrictions, whereas markers 4 and 8 highlight periods of increased N1 gene copy concentrations in campus wastewater. During these times, the research team engaged with campus health and safety teams, alerting them to rising trends in wastewater, which were believed to be indicative of a rise in increased viral shedding on campus. The increase in wastewater viral load was observed without a corresponding concomitant increase to reported cases from campus clinical testing – confirming that the campus population was undertested throughout the pandemic (Fig. 2). Throughout the study, various factors such as changes in restrictions, dominant VoC, campus occupancy, and vaccination levels (Table 1) were considered to collectively influence shifts in campus infections and, consequently, variations in wastewater N1 gene copy concentrations. For instance, marker 7 (Fig. 2) corresponds to a relaxation in both national and local lockdown measures and is linked with a significant increase in SARS-CoV-2 loading, from 2.42 × 10^3^ GC/L on 18/05/2021 to 4.42 × 10^5^ GC/L on 02/06/2021. Here, concordance between positive detection of SARS-CoV-2 in the wastewater and cases on the campus was identified. There were 82 days where there was at least one positive case on the campus, 56.1 % corresponded with a positive detection of SARS-CoV-2 in the wastewater at the WWTW Inlet in the preceding 7 days (95 % CI: 45.2–67.1), 69.5 % corresponded with a positive detection of SARS-CoV-2 in the wastewater at the WWTW Inlet in the preceding 14 previous days (CI: 59.3–79.3) and 92.7 % corresponded with a positive detected at the WWTW Inlet in the preceding 30 days (CI: 86.8–97.6). WBS was thus effective in detecting shifts in virus prevalence on campus. This includes identifying asymptomatic cases not always evident through clinical testing. Findings indicate that wastewater data can act as an early indicator of outbreaks, independent of clinical case reports, emphasizing its importance in public health strategy. Adjustments in restrictions, virus variants, campus occupancy, and vaccination rates influence virus spread, with significant increases in viral load following the easing of lockdown measures. The work herein supports the findings of Fielding-Miller et al. (2023) who reported that 76 % of positive cases in a school corresponded with positive detection of SARS-CoV-2 in their wastewater samples (95 % CI: 68 –75 %), and Karthikeyan et al. (2021b) who found that 84.5 % of positive cases on the University of California San Diego campus corresponded with positive detection of SARS-CoV-2 in their wastewater samples one week prior to sample collection. This would appear to support earlier findings that the wastewater SARS-CoV-2 data can be a leading indicator of SARS-CoV-2 infections and within university campus (Peccia et al., 2020). This study was able to positively identify two of 13 samples that were sequenced, both of which were closely aligned to the Alpha VoC. This was the dominant VoC in the surrounding area at the time. Other researchers have identified SARS-CoV-2 variants at the Near-Source level (Karthikeyan et al., 2021a; Vo et al., 2022), within sewer (Rios et al., 2021) and at the WWTW level (Crits-Christoph et al., 2021; Rios et al., 2021). These results underscore the critical role of wastewater surveillance in informing and guiding pandemic response efforts. Next, we examined any links between wastewater constituents and viral load.

### 3.2 SARS-CoV-2 N1 and its relationship to wastewater constituents

This study offers a detailed source-to-sink apportionment of SARS-CoV-2, supported by a robust dataset on wastewater parameters, both spatially and temporally. Municipal wastewater contains ammonium and phosphate due to urine and faeces, as well as runoff, chemicals, industry, and food waste (Rose et al., 2015; Been et al., 2014). SARS-CoV-2, detected through the N1 gene, is primarily shed in faeces, with a meta-analysis suggesting adults shed the virus for an average of 23.2 days (95 % CI: 19.0–28.4) (Foladori et al., 2020; Yan et al., 2021), which was shorter than the 30 days observed here which provided the strongest correlation. The TSS and tCOD in wastewater represent non-dissolved solid matter and the oxygen required for the chemical oxidation of organic matter in wastewater, respectively (Hu and Grasso, 2005). The results of Spearman's Rank-Order correlation analysis between SARS-CoV-2 and wastewater constituents (Supplementary Information 1), revealed strong positive correlation between SARS-CoV-2 N1 GC concentration and NH_4__—_N in wastewater at the Near-Source level (Spearman's Rank; ρ(14) = 0.823, *p* < 0.01), TSS (Spearman's Rank; ρ(14) = 0.572, *p* < 0.01), and tCOD (Spearman's Rank; ρ(14) = 0.765, *p* < 0.01). However, PO_4_-P did not show a consistent correlation with SARS-CoV-2 at the building, sewer, or WWTW level, possibly due to influences from large washing facilities in Halls of Residence and other sources of PO_4-_P. Some contaminants found in wastewater, i.e., surfactant concentration, disproportionately influences recovery of SARS-CoV-2 genetic material (Kevill et al., 2022), alongside heavy metals and biological activity. This may in part explain the poor correlation between some wastewater parameters (e.g., PO_4_-P) which are easier to measure. Other factors, such as variable flow and sampling regime could impact the strength and significance of the relationship between wastewater constituent and SARS-CoV-2 gene copies. Ingress of ground and/or soil water into the sewer and in-network characteristics can additionally influence results (Wade et al., 2022) although are less likely within this specific case study due to the small scale, isolated, single source aspects of the wastewater conveyance system. Within the Sewer samples, the SARS-CoV-2 N1 GC concentration was positively correlated with NH_4__—_N, tCOD and TSS but to a less extent (evidenced by lower ρ values; Tables 1 and 2 in Supplementary Information 1), suggesting dilution and decoupling between the virus target (SARS-CoV-2) and wastewater constituents.

At the WWTW level, a weak positive correlation was observed between PMMoV normalized SARS-CoV-2 N1 GC concentration and ammonia (Spearman's Rank; ρ(29) = 0.400, *p* = 0.03). This finding highlights two important aspects: firstly, ammonia could be a useful normalization parameter in WWTWs not influenced by industrial inputs; and secondly, the alignment of trends between a viral marker (PMMoV) and a chemical marker (ammonia) suggests their utility as indicators of population levels. It is recommended to use PMMoV in municipal waters for its reliability, while ammonia offers a more rapid and cost-effective option for monitoring at the Near-Source level. Future research should explore the relationship between faecal shedding and the impact of COVID-19 infections on wastewater's chemical and biological markers. Using biomarkers and chemical markers for population normalization in wastewater surveillance faces challenges, including individual variability in excretion rates, environmental degradation affecting marker stability, and potential contamination from non-human sources. Additionally, the lack of standardization across different markers complicates the accurate interpretation of population-level data from wastewater analyses.

**Fig. 3:**
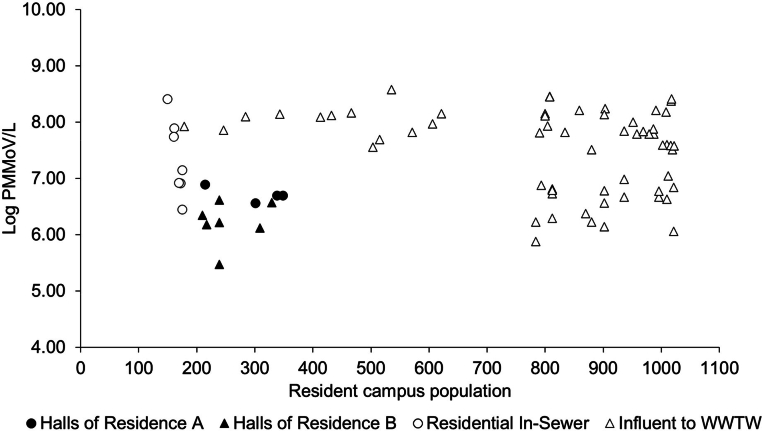
Concentration of PMMoV at different sewershed sample points under varying population.

In samples positive for SARS-CoV-2, PMMoV concentrations varied from 1.07 × 10^5^ to 6.41 × 10^8^ GC/L (Fig. 3), without significant correlations to wastewater constituents near the source. However, a positive correlation was noted between PMMoV concentrations and tCOD at the In-Sewer level (Spearman's Rank; ρ(24) = 0.490, *p* = 0.046), whereas no significant correlation was found between PMMoV and SARS-CoV-2 N1 concentrations. Interestingly, at the WWTW Influent, PMMoV negatively correlated with TSS (Spearman's Rank; ρ(97) = -0.245, *p* = 0.016), with no other significant relationships detected. PMMoV levels at the WWTW Influent matched the ranges reported by Symonds et al. (2018) as the lower quartile concentration of PMMoV was 7.56 × 10^6^ GC/L, the median value was 3.97 × 10^7^ GC/L, the upper quartile value was 8.97 × 10^7^ GC/L with an interquartile range of 8.21 × 10^7^ GC/L. The observed lack of correlation between PMMoV and SARS-CoV-2 N1 concentrations in the context of on-campus populations, particularly in settings with limited COVID-19 infections, reveals a nuanced aspect of WBS. This decoupling between the viral tracer (PMMoV) and the pathogen of interest (SARS-CoV-2) underscores the complexity of using environmental surveillance as a tool for tracking infectious diseases within small populations. It suggests that while PMMoV serves as a reliable indicator of general viral presence within a larger community, its utility may diminish in more localized or constrained settings where the incidence of COVID-19 is low. This is likely due to the inherent variability in shedding rates, the impact of dilution effects, and the presence of competing or confounding biological and chemical substances within the wastewater matrix.

**Fig. 4:**
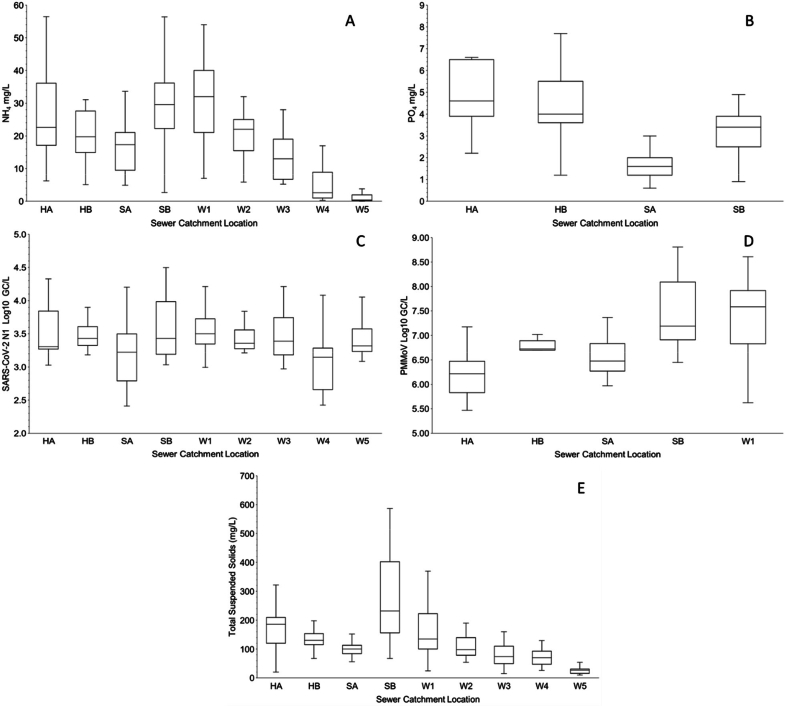
A) NH_4__—_N mg *L*^−1^, B) PO_4_-P mg *L*^−1^, C) SARS-CoV-2 N1 Log10 GC/*L*, D) PMMoV Log10 GC/*L* and E) TSS mg *L*^−1^ at sewershed sample points. N.B: HA – Halls of Residence A, HB – Halls of Residence B, SA – Technical In-Sewer, SB – Residential In-Sewer, W1 – Influent, W2 – Post Primary Lamella, W3 – Post Roughing Trickling Filter, W4 – Post Secondary Trickling Filter and W5 – Final Effluent.

Fig. 4 provides an overview of the wastewater constituents from source to sink, highlighting the variability in NH_4__—_N concentrations with median values of 22.6 mg/L for Halls A and 19.7 mg/L for Halls B. NH_4__—_N concentrations peaked at 32 mg/L at the WWTW inlet but were reduced to 2.6 mg/L following secondary treatment processes. In contrast, PO_4_-P showed higher median concentrations in Halls A and B (4.6 and 4.0 mg/L, respectively), which became more diluted in the sewer, ranging from 1.6 to 3.4 mg/L. The variability in SARS-CoV-2 abundance across the campus reflects the dynamics of infection and faecal shedding, with average log N1 GC/L values of 3.37 for Halls, 3.33 for the sewer, and 3.34 for all WWTW samples. PMMoV abundance, indicating a rising population equivalent across sample locations and through the conveyance system, showed median log GC/L values increasing from 6.22 to 6.72 in Halls A and B to 6.82 in the sewer and further to 7.58 in the WWTW inlet. TSS concentrations varied considerably, with Halls A and B recording 186 mg/L and 131 mg/L, respectively; the technical in-sewer had the lowest median at 100 mg/L, while the residential in-sewer had the highest at 232 mg/L. The WWTW inlet showed a median TSS of 135 mg/L, with values decreasing through the treatment process to between 98 and 27 mg/L from the post-primary lamella to the final effluent.

In summary, the variations in wastewater constituents from source to sink, demonstrate the impact of human activities and wastewater treatment efficiency. NH_4__—_N and PO_4_-P concentrations vary, but NH_4__—_N significantly decreases after secondary treatment, demonstrating treatment effectiveness. SARS-CoV-2 variability indicates faecal shedding and reflects infection trends, while PMMoV increases from residential areas to the WWTW inlet, highlighting value for estimating population scale. These results underscore wastewater's complexity for environmental surveillance in a relatively simple conveyance and treatment works. As the scale and complexity of the sewer system expand, there is a need for increased sampling frequency and enhanced sample resolution to fully elucidate trends for public health monitoring.

### 3.3 Removal of SARS-CoV-2 at on campus WWTW

The efficacy of SARS-CoV-2 removal in wastewater treatment works (WWTW) has been explored in prior studies (Foladori et al. 2020). Randazzo et al. (2020) observed a low detection rate of SARS-CoV-2 post-secondary treatment (11 %, or 2 out of 18 samples), despite its presence in 83 % of influent samples (35 out of 42). Given the hypothesis that SARS-CoV-2 binds to particulates in wastewater, we analysed the removal efficiency of TSS and SARS-CoV-2 genetic material throughout the treatment stages at the WWTW. Utilizing the Kruskal–Wallis 1-way ANOVA test, we identified a statistically significant difference in daily TSS daily loadings across the WWTW stages (*p* < 0.01), indicating effective TSS removal. Post-hoc Dunn's test identified that there was a statistically significant difference in TSS concentrations between the Post-Secondary Trickling Filter and the Final Effluent stages (*p* < 0.01), indicating effective removal of solids between these two stages of wastewater treatment. Conversely, no significant difference was found in the daily loadings of N1 gene copies across treatment stages (*p* > 0.05), suggesting that the WWTW did not effectively remove SARS-CoV-2, contrasting with findings from Randazzo et al. (2020). This discrepancy could be attributed to differences in treatment processes between the studies, highlighting the need for further research into the behaviour of enveloped viruses like SARS-CoV-2 in various WWTW configurations.

### 3.4 Implications for wastewater monitoring studies

This study on a university campus in England highlights the potential of WBS as an effective, non-invasive tool to monitor and manage public health, particularly in densely populated settings like university campuses (Ng et al., 2024). The detection of SARS-CoV-2 and its variants (Boehm et al., 2022) in wastewater provides a unique opportunity for early identification of infections and tracking the spread of the virus, even before clinical cases are confirmed (Betancourt et al., 2021). This preemptive approach can inform and guide public health decisions, allowing for timely interventions such as targeted testing, isolation measures, and adjustments to campus operations to prevent outbreaks (Hassard et al., 2021, 2023). Moreover, the study underscores the importance of understanding the dynamics of viral load in wastewater at different stages, from source to treatment. The correlation between specific wastewater constituents and SARS-CoV-2 levels can offer insights into the efficacy of treatment processes and potential risks to the environment and public health (Wu et al., 2024).

For university campuses, integrating WBS with clinical testing can create a more robust surveillance framework, enhancing the ability to monitor and respond to infectious diseases (Kevill et al., 2024). This integration can help optimize resource allocation, prioritize testing and vaccination efforts, and tailor public health strategies to the unique context of each campus to minimize testing burden whilst protecting public health (Amirali et al., 2024). Future efforts should focus on refining sampling and analytical methods to improve the sensitivity and specificity of WBS, exploring the use of additional biomarkers, and developing standardized protocols for data interpretation and reporting. By advancing our understanding and application of WBS, universities can enhance their preparedness and resilience against not only COVID-19 but also other infectious diseases that may pose threats in the future.

## 4 Conclusions

In conclusion, this work utilised WBS to track the dynamics of SARS-CoV-2 across a university campus, leveraging a comprehensive WBS approach to offer insights into the virus's spread, from source (e.g., groups of buildings) to the sink (environmental discharge). Initial findings revealed significant fluctuations in SARS-CoV-2 levels in wastewater, aligning with the enforcement and easing of local and national restrictions, underscoring the method's potential as an early warning system – although applied retrospectively here. The work notably identified the presence of VoCs, with shifts in viral prevalence aligning with changes in campus and community infection rates, despite the challenges posed by variable detection rates by clinical testing.

Further analysis of wastewater constituents highlighted the complex interplay between biological markers like PMMoV and chemical makers such as ammonium and phosphate, emphasizing the need for enhanced sampling frequency and resolution to accurately monitor viral loads in more complex sewer systems. The study's findings underscore the critical role of WBS in public health strategies, demonstrating its utility in identifying asymptomatic cases and monitoring shifts in viral prevalence independently of clinical case reports. However, the work acknowledges the limitations inherent in WBS, including challenges related to sample storage, contamination, dilution effects, and underrepresented clinical data. Despite these hurdles, the evidence presented supports the role of WBS as a valuable adjunct to traditional public health surveillance, highlighting its applicability in university settings for tracking infections and informing targeted public health interventions.

This research contributes to the growing body of knowledge on the efficacy of WBS in environmental monitoring and infectious disease surveillance, calling for further investigation to refine methodologies and improve our understanding of viral behaviour in wastewater systems. By enhancing our ability to detect and respond to infectious disease outbreaks, WBS can play a pivotal role in safeguarding public health, particularly in densely populated or geographically isolated communities.

## CRediT authorship contribution statement

**M. Folkes:** Formal analysis, Data curation, Writing – original draft, Visualization. **V.M. Castro-Gutierrez:** Formal analysis, Visualization. **L. Lundy:** Writing – review & editing, Funding acquisition. **Y. Bajón-Fernández:** Writing – review & editing. **A. Soares:** Supervision, Writing – review & editing. **P. Jeffrey:** Supervision, Writing – review & editing, Funding acquisition. **F. Hassard:** Conceptualization, Methodology, Investigation, Writing – review & editing, Supervision, Project administration, Funding acquisition.

## Declaration of competing interest

The authors declare that they have no known competing financial interests or personal relationships that could have appeared to influence the work reported in this paper. The decision to publish the research rested solely with the authors and the funder did not influence the decision to publish the research.

## Data Availability

Metadata supporting this study are not publicly available due to potential ethical considerations. However, data can be made available on request. Please contact francis.hassard@cranfield.ac.uk.

## Appendix Supplementary materials

Image, application 1
