# Supplementary material for: Campus source to sink wastewater surveillance of severe acute respiratory syndrome coronavirus-2 (SARS-CoV-2)
Source: Curr Res Microb Sci. 2024 May 6;6:100240. doi: 10.1016/j.crmicr.2024.100240 (PMC11106825; doi:10.1016/j.crmicr.2024.100240)
Supplement: Supplementary file 1 [file mmc1.docx]

**Supplementary Material 1**

RNA extraction Protocols:

Protocol 1:

The 250ml sample was aliquoted into 4 × 50 ml falcon tubes, which were centrifuged for 30 minutes at 3,000 xg at 4°C, and the supernatant transferred to 250 ml PCCO centrifuge bottles (Thermo Fisher, UK). Supernatants were spiked with a surrogate non-enveloped virus (murine norovirus) for extraction control before concentration with polyethylene glycol (PEG) precipitation, with incubation in a refrigerator at 4°C for at least 12 hours. The samples were then centrifuged at 10,000 ×g for 30 minutes at 4°C, excess PEG was discarded by pouring, followed by further centrifugation at 10,000 xg for 10 minutes at 4°C to form a final concentrated pellet. Excess supernatant was once again discarded. This pellet was then re-suspended in 0.5 mL of molecular biology grade phosphate buffered saline (PBS), pH 7.4. RNA extractions from the pellets were then conducted using the NUCLEISENS® RNA extraction kit on a MINIMAG® (BioMérieux, France).

Protocol 2:

Protocol 2 was adapted from Amirouche et al. (2021). Samples were extracted in duplicate where possible. The falcon tubes were centrifuged for 30 minutes at 3000 ×g at 4°C, and the supernatant transferred to 250 ml PCCO centrifuge bottles (Thermo Fisher, UK). Supernatants were spiked with a surrogate enveloped virus (Φ6) for extraction control before concentration with polyethylene glycol (PEG) precipitation. Samples were shaken at 200 rpm on an orbital shaker for 15 minutes at room temperature, prior to incubation in a refrigerator at 4°C for at least 2 hours. A pilot study on 3 wastewater samples extracted in triplicate revealed that between 2-24 hours of incubation did not impact the RNA yield or RNA quality. No significant difference was found between samples incubated for 2 - 24 hours. The samples were then centrifuged at 10,000 ×g for 30 minutes at 4°C, excess PEG was discarded by pouring, followed by further centrifugation at 10,000 ×g for 10 minutes at 4°C to form a final concentrated pellet. Excess supernatant was discarded, with 1 mL retained. This was used to resuspend the pellet prior to transfer to a 1.5 mL microcentrifuge tube. The samples were then centrifuged at 12,500 ×g for 5 minutes, re-forming the pellet. The supernatant was then removed by pipetting.

The pellet was resuspended in 800 μL of TRIzol™ (Fisher Scientific, UK) by pipetting and vortexing to lyse the sample. 200 µL of Chloroform (Fisher Scientific, UK) was then added, and mixed by vortexing. The samples were incubated at room temperature for 3 minutes. The sample was then centrifuged at 12,000 ×g for 15 minutes at 4°C. The aqueous layer containing the RNA was then transferred to sterile, RNAase free Lo-Bind Eppendorf tubes (Fisher Scientific, UK). RNA was then extracted and purified using a Macherey Nagel Nucleospin RNA kit (Fisher Scientific, UK)

**SARS-CoV-2 N1 and its relationship to wastewater constituents**

Table 1: Spearman rank-order correlations between SARS-CoV-2 N1 and E gene copies and wastewater constituents for Halls of Residence A and B. N.B: * significant to the 0.05 level, ** significant to the 0.01 level.

| Wastewater Constituent (mg/L) | Target Gene (GC L^-1^) | |
| --- | --- | --- |
|  | Nucleocapsid Region 1 (N1) | Envelope Protein (E) |
| NH_4_^+^ | 0.823**  N = 16 | 0.537**  N = 19 |
| PO_4_^2-^ | 0.466  N = 16 | 0.372  N = 18 |
| TSS | 0.572**  N = 16 | 0.376  N = 18 |
| tCOD | 0.765**  N = 16 | 0.315  N = 15 |

Table 2: Spearman rank-order correlations between SARS-CoV-2 N1 and E gene copies and wastewater constituents for Technical and Residential In-Sewer sample points. N.B: * significant to the 0.05 level, ** significant to the 0.01 level.

| Wastewater Constituent | Target Gene (GC L^-1^) | |
| --- | --- | --- |
|  | Nucleocapsid Region 1 (N1) | Envelope Protein (E) |
| NH_4_^+^ | 0.543**  N = 28 | 0.293  N = 19 |
| PO_4_^2-^ | 0.342  N = 25 | -0.130  N = 18 |
| TSS | 0.408*  N = 29 | 0.381  N = 22 |
| tCOD | 0.528**  N = 27 | 0.407  N = 21 |
